# Exploring Imaging Depth: A Pilot Study About 2D vs. 4D Echocardiography for Tricuspid Valve Evaluation

**DOI:** 10.31083/RCM41556

**Published:** 2025-05-19

**Authors:** Giuseppe Santarpino, Giovanni Taverna, Vincenzo Calabrese, Flavia Coviello, Giancarlo Trimarchi, Olimpia Trio, Corrado Fiore, Giuseppe Andò, Giuseppe Nasso, Giuseppe Speziale

**Affiliations:** ^1^Department of Cardiac Surgery, Città di Lecce Hospital, GVM Care & Research, 73100 Lecce, Italy; ^2^Department of Clinical and Experimental Medicine, Magna Graecia University, 88100 Catanzaro, Italy; ^3^Department of Cardiac Surgery, Paracelsus Medical University, 90419 Nuremberg, Germany; ^4^Department of Clinical and Experimental Medicine, University of Messina, 98124 Messina, Italy; ^5^Department of Medicine and Surgery, University of Enna, 94100 Enna, Italy; ^6^Interdisciplinary Center for Health Sciences, Scuola Superiore Sant’Anna, 56127 Pisa, Italy; ^7^Department of Cardiology, Città di Lecce Hospital, GVM Care & Research, 73100 Lecce, Italy; ^8^Department of Cardiac Surgery, Anthea Hospital, GVM Care & Research, 70124 Bari, Italy

**Keywords:** 4D echocardiography, tricuspid valve, tricuspid annulus

## Abstract

**Background:**

The tricuspid valve (TV) is a complex three-dimensional (3D) anatomical structure; however, current guidelines recommend tricuspid annulus (TA) measurements to be performed with two-dimensional (2D) echocardiography. The aim of this study was to compare TV measurements obtained with 2D and four-dimensional (4D) echocardiography for surgical planning.

**Methods:**

All echocardiographic data of patients referred to our center for TV assessment were collected. Multimodality imaging data were reviewed, including 2D transthoracic echocardiography (TTE) integrated with information from 3D TTE. Measurements were also compared with those obtained using the 4D Auto Tricuspid Valve Quantification (TVQ) tool.

**Results:**

Overall, 11 patients (median age 72 [66–78] years, 18% female) were included in the study. Mild, moderate and severe tricuspid regurgitation (TR) was present in 6, 3 and 2 patients, respectively. Systolic pulmonary artery pressure was 35 ± 8 mmHg, inferior vena cava diameter 21 ± 4 mm, right atrial area 25 ± 9 cm^2^, 4D ejection fraction 45 ± 7%, 4D fractional area change 40 ± 6%, and tricuspid annular plane systolic excursion 21 [15–25] mm. 2D/4D right ventricular-basal diameter (RVD1) was significantly different (*p* < 0.005). Similarly, 2D/4D right ventricular diameter measured at the level of the left ventricular papillary muscles (RVD2) was significantly different (*p* < 0.012), as well as 2D/4D tricuspid annular diameter (*p* = 0.020). Despite these differences, a strong correlation between variables was observed (Spearman correlation coefficient >0.824). In evaluating the correlation between TR severity and analyzed variables, RVD1 was related to TR severity both at 2D and 4D echocardiography. Conversely, RVD2 and TA diameter were significantly associated with TR severity only at 4D echocardiography.

**Conclusions:**

Our results suggest that specific patient subsets could benefit more from TA measurements using the 4D Auto TVQ tool to help identify the mechanisms responsible for TR, including candidates for left-sided valve surgery and patients in whom the indication for TV repair is unclear.

## 1. Introduction

The tricuspid valve (TV) is a complex three-dimensional (3D) anatomical structure, involving various components of the right heart. It is the largest valve of the human heart, with a saddle-shaped elliptical annulus and an annular area ranging from 9.72 ± 2.08 cm^2^ to 9.94 ± 2.33 cm^2^ [1, 2], increasing significantly during atrial systole and end-diastole, as does its circumference. According to current guidelines, tricuspid annulus (TA) diameter should be measured using two-dimensional (2D) echocardiography from the apical 4-chamber view during diastole. Normal TA diameter in adults is 28 ± 5 mm, and significant dilatation is defined by an end-diastolic diameter >21 mm/m^2^. An end-systolic diameter >32 mm or an end-diastolic diameter >34 mm are usually associated with significant tricuspid regurgitation (TR) [3].

Numerous conditions may impact valve function and size, particularly volume load, therefore the assessment for the presence of severe TR is challenging. In addition, echocardiographic quantification is not accurate as it is affected by right ventricular (RV) preload, afterload, and function.

The prevalence of severe TR is ~10–15% in patients undergoing left-sided valve surgery. According to current guidelines, TV surgery should also be considered in patients with mild or moderate TR with a dilated annulus (≥40 mm or >21 mm/m^2^) by 2D echocardiography [4]. This underscores the relevance of 2D echocardiographic annular measurements, as ultrasound assessment may be more affected by RV filling, though 2D measurement of a 3D saddle-shaped structure represents *per se* a technical compromise.

The aim of this study was to compare 2D vs 4D echocardiographic measurements of the TV using a four-dimensional (4D) Auto Tricuspid Valve Quantification (TVQ) tool (GE Healthcare Vingmed, Horten, Norway) in order to provide more accurate information for surgical planning of TV repair.

## 2. Methods

From January to April 2024, all echocardiographic data of patients referred to the Città di Lecce Hospital, GVM Care & Research for TV assessment were collected at baseline. All echocardiographic data sets were acquired using a GE Vivid E95 ultrasound system (GE Healthcare; Vingmed Ultrasound, Horten, Norway) equipped with an M5S probe (frequency range: 1.5–4.6 MHz; GE Healthcare; Vingmed Ultrasound, Horten, Norway) and the 4Vc-D matrix-array transducer (5692036, GE Healthcare; Vingmed Ultrasound, Horten, Norway). Video loops and images (2D–4D) were stored in the morning and analyzed in the afternoon during medical reporting by an expert cardiologist with 10 years of professional experience in echocardiography. All the measurements were performed again by trained fellows in echocardiography blinded to clinical data. Intraobserver variability was assessed by the same expert cardiologist one month later. While the interobserver variability was assessed by 2 independent blinded cardiologists (fellows in echocardiography) in a randomly selected subgroup of patients.

Exclusion criteria were active cancer, pregnant women, congenital heart disease, atrial fibrillation, renal or hepatic failure, inadequate image quality. Inclusion criteria were age >17 years, good-quality echocardiographic images, left-sided valve surgery. All patients were examined in the left lateral decubitus position. RV-focused apical 4-chamber views were acquired for the best visualization of the TV apparatus. Due to the complex anatomy, TV measurements were performed with multi-modality imaging, including 2D transthoracic echocardiography (TTE) integrated with 3D TTE [5, 6]. Real-time three-dimensional echocardiography (3DE), also named four-dimensional echocardiography (4DE), is a new method where the new dimension is time. All three-dimensional images are simultaneously in real time and in motion. The guidelines of the American Society of Echocardiography recommend TA sizing by two-dimensional transthoracic echocardiography (2DE) [4, 7]. However, TA has a saddle-shaped morphology and, because of this three-dimensional (3D) architecture, only a single linear dimension isn’t enough for its actual size [8, 9]. Moreover, the TA dilation is typically in the anteroposterior direction, which is not explored in the 2DE apical 4-chamber view [10]. So 3D/4D is a more accurate method to assess TV anatomy than 2DE, but nowadays this approach is technically difficult [11]. Indeed, many cardiology centers don’t have software for 4D quantification, so only the use of echo 2D is still a common use in general hospital clinical practice.

For the quantification of TV morphology, the novel 4D Auto TVQ tool was used. This tool is designed for clarifying the main mechanism (i.e., annulus dilation, leaflet tethering or mixed) in patients with functional tricuspid regurgitation (FTR) mostly if still unclear by 2DE. It’s useful too in patients with FTR who candidates for left-sided valve surgery are, device implantation or in patients who are considered for catheter-based interventional repair procedures. In these patients the quantification of TV morphology is mandatory, mostly when the indication to perform TV repair is unclear.

**Fig. 1.**
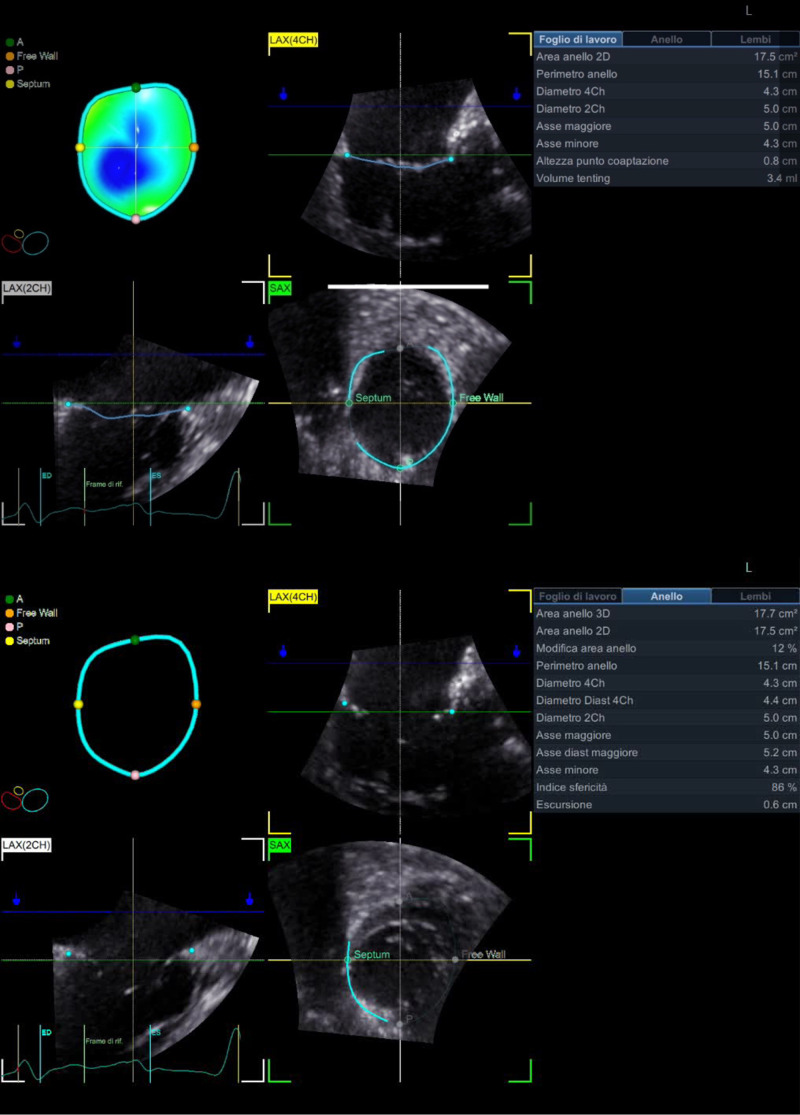
**Tricuspid valve measurements using the 4D Auto TVQ analysis tool**. Three-dimensional TV annulus model in patients with functional tricuspid regurgitation (FTR) seen en-face from the ventricular perspective after setting landmarks of TV on 4CH, 2CH and then on SAX. Quantitative analysis of the tricuspid annulus geometry: 2D area, 3D area, area change, perimeter, 4CH diameter, 4CH diastolic diameter, 2CH diameter, major axis, major diastolic axis, minor axis, sphericity index, excursion. Leaflets parameters: coaptation point height, max tenting height, tenting volume. 4D, four-dimensional; TVQ, Tricuspid Valve Quantification; TV, tricuspid valve; 4CH, 4 chamber; 2CH, 2 chamber; SAX, short-axis view; 2D, two-dimensional; 3D, three-dimensional; LAX (2CH), long axis 2 chamber.

This tool enables a rapid semi-automated detection of the TV leaflet surface in only one systolic frame during the cardiac cycle. Quantitative TV measurements analysis included: annulus parameters (3D area, 2D area, perimeter, 4CH diameter, 2CH diameter, major diameter, minor diameter, sphericity index, excursion) and leaflets parameters (Coaptation point Height, Max Tenting Height, Tenting Volume) (Fig. 1).

It’s specifically designed for the quantification of tricuspid valve (TV) morphology, but it’s not yet validated against cardiac magnetic resonance imaging (MRI). It’s only verified against manual multiplanar reconstruction (MPR) measurements, anyway clinical studies are ongoing in this field.

**Fig. 2.**
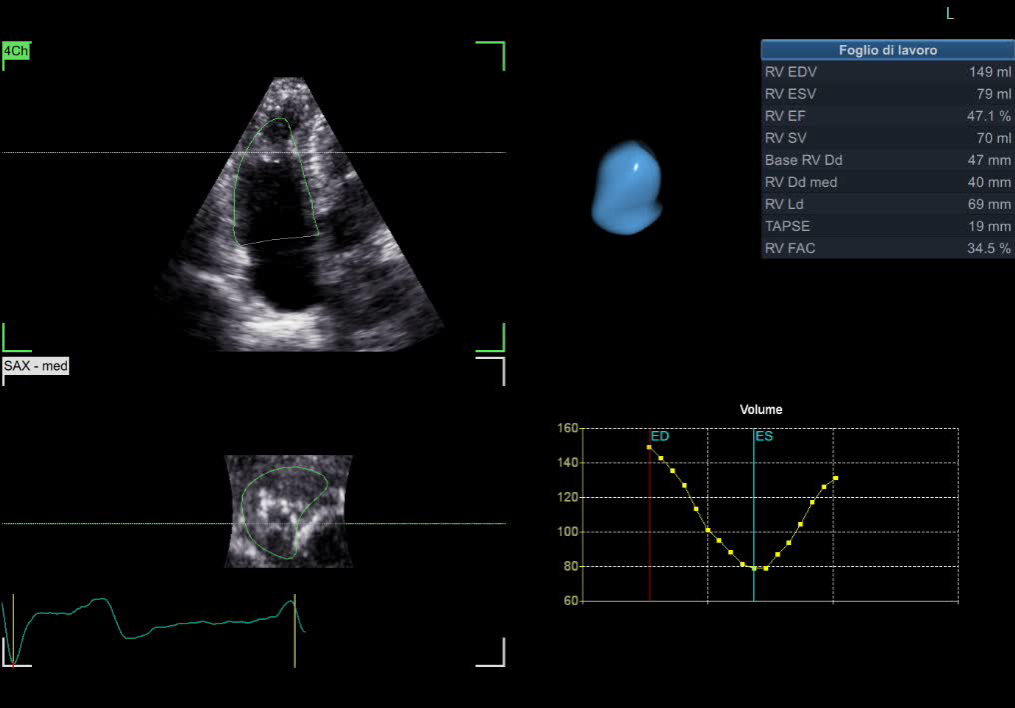
**Right ventricle measurements using the 4D RVQ tool**. Three-dimensional right ventricular model and worksheet after using 4D RVQ tool for quantification. The contours can easily be edited by the operator after placed six landmark points. The worksheet and a time–volume curve are visualized at the results stage. EDV, end-diastolic volume; ESV, end-systolic volume; EF, ejection fraction; SV, stroke volume; RV, right ventricular; Dd med, diameter (medium-level); Ld, diameter (lower); TAPSE, tricuspid annular plane systolic excursion; FAC, fractional area change; RVQ, right ventricle quantification.

The RV 3D model and worksheet were obtained using the 4D Auto right ventricle quantification (RVQ) tool. This tool is easily used by the operator for editing the contours after placement of six landmark points (two TA points and the RV apex point in the 4CH view, and the RV/LV posterior and anterior points plus the RV free wall point in the SAX mid papillary view). The worksheet and a time–volume curve were visualized at the results stage (Fig. 2).

Additional information on the mechanisms underlying TV dysfunction leading to altered global RV kinetics was derived from tissue Doppler and color Doppler data. The anterior and septal leaflets were visualized on the TTE apical 4-chamber view, whereas the posterior leaflet was identified more reliably in the apical 2-chamber view, since the plane passes through the lower part of the RV free wall adjacent to the diaphragm.

During the echocardiographic examination, several indices were obtained to evaluate TV function and morphology, including ​​TR area allowing an estimation of TR severity and the tricuspid annular plane systolic excursion (TAPSE). Additionally, the peak systolic pressure gradient between the right atrium and right ventricle was measured as it is an important hemodynamic marker for evaluating pulmonary artery pressure and RV function. The assessment of RV size by means of 2D and 3D echocardiography included measurement of RV diameter and right atrial area using RV-focused apical 4-chamber views. RV size is best estimated at end-diastole [5]. Assessment of RV systolic function was also performed using several parameters, including TAPSE and fractional area change (FAC) on 2D echocardiography, which have been found to be significant markers of RV contractile function [6], and RV ejection fraction on 2D and 3D echocardiography, longitudinal strain and strain rate parameters.

All patients gave written informed consent for use of their clinical data, and data collection, analysis, publication and storage were approved by the ethics committee (BE-CdLH 1/24).

### Statistical Analysis

All variables were not normally distributed at the Shapiro-Wilk test, and data are reported as median and interquartile range. Differences in a same variable obtained using different methods (i.e., 2D/4D RV-basal diameter [RVD1], 2D/4D RV diameter measured at the level of the left ventricular papillary muscles [RVD2], and 2D/4D TA diameter) were detected by Wilcoxon test for dependent variables. The exact Fisher test has been applied where appropriate. Spearman correlation test was used to compute correlation analysis. Needed sample size was not computed due to this analysis was considered as Pivotal study.

## 3. Results

Overall, 11 patients (median age 72 [66–78] years, 18% female) were included in the study. We included only 11 patients with good-quality echocardiographic images who underwent cardiac surgery in our center.

All patients had symptoms of dyspnea (NYHA [New York Heart Association] class II in 8 patients and NYHA class III in 3 patients). Mild, moderate, and severe tricuspid regurgitation (TR) was present in 6, 3 and 2 patients, respectively. The etiology for TR in all patients was Secondary or Functional TR caused by dilatation of the TV annulus related to right ventricular or right atrial remodeling. Systolic pulmonary artery pressure (sPAP) was 35 ± 8 mmHg, inferior vena cava diameter 22 [18–23] mm, right atrial area 25 [17–28] cm^2^, 4D ejection fraction 45 [39–51], 4D FAC 41 [35–46], and TAPSE 21 [15–25] mm.

**Fig. 3.**
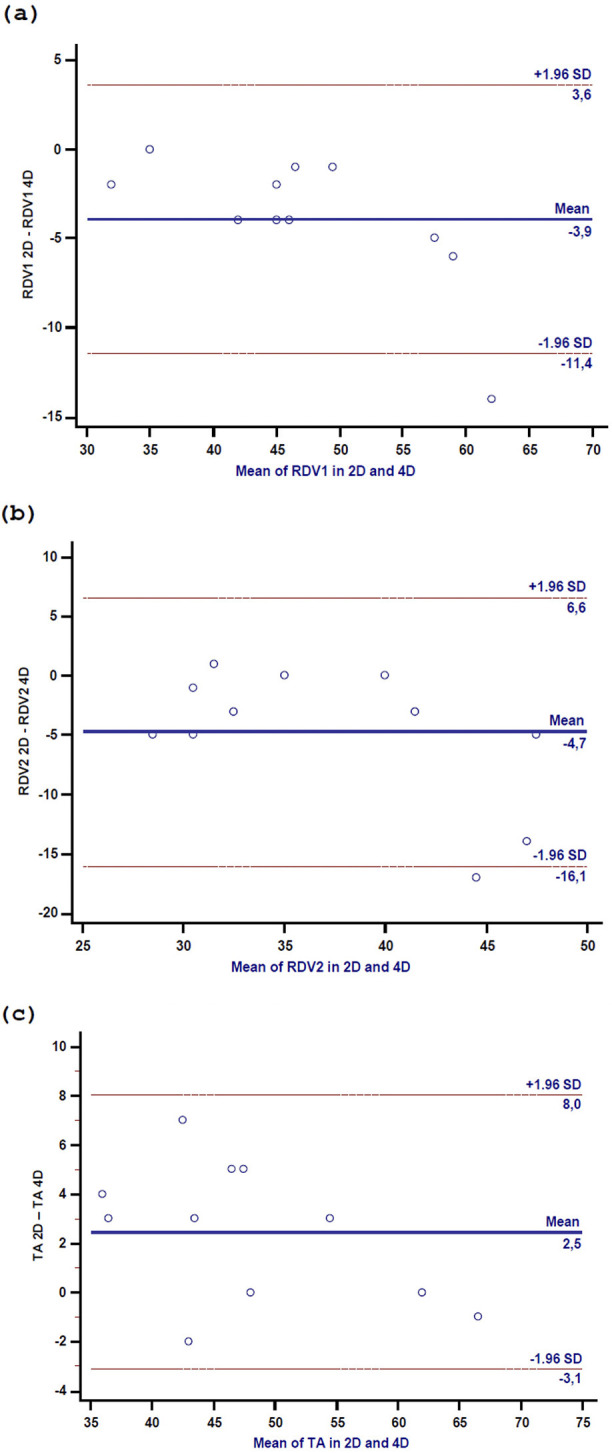
**Scatter plot in 2D and 4D of RDV1 (a), RDV2 (b), and TA diameter (c)**. RVD1, right ventricular-basal diameter; RVD2, right ventricular diameter measured at the level of the left ventricular papillary muscles; TA, tricuspid annulus.

Significant differences were observed between 2D and 4D measurements for RDV1 (right ventricular-basal diameter), RDV2 (right ventricular diameter measured at the level of the left ventricular papillary muscles), and TA diameter (Table 1), particularly for 2D vs. 4D RVD1 (2D RDV1 44 [40–55] vs 4D RDV1 47 [44–60]; *p* < 0.005) (Fig. 3a).

**Table 1.** **Differences between 2D and 4D measurements for RVD1, RVD2 and TA diameter**.

	2D	4D	*p*-value
RVD1 (mm)	44 [40–55]	47 [44–60]	0.005
RVD2 (mm)	35 [30–40]	35 [31–50]	0.012
TA diameter (mm)	48 [42–56]	44 [39–53]	0.020

Values are reported as median [interquartile range].

Similarly, a significant difference was found between 2D vs. 4D RVD2 (2D RDV2 35 [30–40] vs 4D RDV2 35 [31–50], *p* < 0.012) (Fig. 3b), as well as 2D vs. 4D TA diameter (*p* = 0.020) (Fig. 3c). Notwithstanding this, a strong correlation between variables was observed (Spearman correlation coefficient of 0.824) (Table 2). Among the variables analyzed, RVD1 was correlated with TR severity both in 2D and 4D measurements (2D RDV1 β = 0.64, *p* = 0.034; 4D RDV1 β = 0.68, *p* = 0.023). Conversely, RVD2 and TA diameter were significantly associated with TR severity only in 4D measurements (4D RDV2 β = 0.61, *p* = 0.047) (Table 3). Although variables were not normally distributed precluding performing the Steiger’s test to compare correlation coefficients, the differences in Spearman coefficients and statistical significance suggest a higher association between TR and 4D measurements rather than TR and 2D measurements.

**Table 2.** **Correlation between 2D vs 4D measurements for RVD1, RVD2 and TA diameter**.

	Spearman r	*p*-value
RVD1 2D vs 4D	0.94	<0.001
RVD2 2D vs 4D	0.82	0.002
TA diameter 2D vs 4D	0.92	<0.001

**Table 3.** **Correlation between 2D and 4D measurements for RVD1, RVD2 and TA diameter and tricuspid regurgitation severity**.

	Spearman r	*p*-value
RVD1 2D	0.64	0.034
RVD1 4D	0.68	0.023
RVD2 2D	0.51	0.107
RVD2 4D	0.61	0.047
TA diameter 2D	0.52	0.098
TA diameter 4D	0.66	0.029

## 4. Discussion

The TV is a complex anatomical structure, with a saddle-shaped elliptical annulus, making manual quantification on 2D echocardiography or 3D multi-planar reconstruction challenging. The tricuspid annulus is also a highly dynamic structure, with various changes in size and shape during cardiac cycle, which are difficult to fully characterize and measure frame-by-frame, unless the use of a novel semi-automated tracking tool, able to follow its changes in three dimensions. As the options for addressing tricuspid insufficiency continue to evolve, including both surgical and percutaneous intervention techniques, the meticulous analysis of annulus diameters and dimensions becomes increasingly essential [12]. This thorough assessment is crucial for planning both surgical and percutaneous procedures, as it significantly influences the procedural outcomes [13, 14].

Therefore, there is the need for a quantification TV tool, such as the 4D Auto TVQ tool. In our pivotal study, we found a significant difference between measurement of RV variables obtained with the 4D Auto TVQ tool vs 2D echocardiography, confirming the importance of a more accurate assessment of the TV. 2D echocardiography is the first-line imaging modality for TV evaluation, though quantitative analysis of the TV apparatus is burdened by limited accuracy. TA diameter by 2D echocardiography systematically underestimates the true largest size of the annulus. In functional TR, TA enlargement occurs predominantly in the antero-posterior direction, and the largest diameter is often not oriented in the septo-lateral direction shown in the 4-chamber view [15, 16]. Only 3D echocardiography allows to acquire the 3D TA area and perimeter mostly for its complex geometry [17]. In clinical routine, echocardiography is the most frequently used imaging technique to assess patients with heart valve diseases [18]. Cardiac magnetic resonance (CMR) and cardiac computed tomography (CCT) are new and emerging imaging modalities but less used in clinical practice for three-dimensional visualization of the TA. Indeed, the limited access in some medical centers which need specific imaging protocols for the right heart, and the use of contrast media or radiation reduce their application mainly to patients undergoing evaluation for TV surgery. In symptomatic patients with severe functional TR, all these imaging variables—TA size, extent of leaflet tenting, right atrial size and RV size and function—are carefully weighted for choosing the best approach or device for valve repair. An antero-posterior TA diameter ≥36 mm and a tenting volume ≥2.30 mL were found to be independent predictors of residual severe TR after surgical TV annuloplasty, with 100% sensitivity and 84% specificity [19]. Thus, for surgical patients, the recognition of severe TV tenting might suggest the surgeon to implement leaflet augmentation techniques during the annuloplasty or valve replacement, as annuloplasty alone might worsen functional TR [20, 21]. According to current guidelines [4], TV surgery is recommended in patients with severe secondary TR undergoing left-sided valve surgery (recommendation class I, level of evidence B). In addition, TV surgery should be considered in patients with severe secondary TR who are symptomatic or have RV dilatation, or in patients with left ventricular dysfunction and pulmonary hypertension.

In reference to these guideline recommendations, a few aspects related to our analysis should be underlined. First, TV surgery has a central role, given that in current guidelines transcatheter procedures should be reserved only for patients not amenable to surgery. In this respect, treatment of TV disease differs greatly from treatment of other valvular pathologies, namely severe aortic stenosis for which transcatheter procedures are preferred over conventional surgery, particularly in some specific age groups (≥75 years). Second, although current guidelines provided an absolute indication for the concomitant treatment of TV disease during mitral valve surgery, evidence derived from surgical databases worldwide shows that TV disease remains undertreated. The reasons why cardiac surgeons very often do not perform concomitant TV surgery deserves further in-depth analysis. Third, even the indication for TR treatment in symptomatic patients, or in those with TA or RV dilatation or pulmonary hypertension (recommendation class IIa) points to the treatment of heart valve disease in most cases. However, many questions remain open regarding mortality and outcome of patients with uncorrected moderate TR and the subsequent evolution of valve disease.

In patients with TA dilatation not treated with annuloplasty and undergoing mitral valve surgery, TR increases by more than 2 grades compared to no or 1 grade increase in patients receiving concomitant tricuspid annuloplasty [22]. Contrary to current beliefs, TR does not disappear spontaneously after correction of left-sided valve disease. These data demonstrate that TA dilatation results in impairment of RV function with progressive and clinically relevant TR. Treatment of mitral lesions only reduces afterload, but it does not correct TA dilatation or affect RV preload or function.

Therefore, combined mitral valve repair and tricuspid annuloplasty should be considered in patients with TA dilatation also in the absence of significant TR. In a study conducted between 2003 and 2011, a strategy of routine treatment of moderate TR and/or significant TA dilatation at the time of mitral valve repair in patients with degenerative mitral regurgitation was associated with freedom from recurrent TR [23]. This above-mentioned study further supports the importance of a 4D echocardiographic tool for better surgical planning. Furthermore, the opportunity to quantify the right ventricle parameters (diameters and area of RV) using 4D Auto RVQ from the same data set of patients on which the 4D Auto TVQ analysis is done, is less time-consuming and provides more precise and reproducible results than the corresponding 2D parameters. The more precise characterization of right-heart structures allows to better understand the central role of RA enlargement in determining TA size in atrial fibrillation (AF) either in healthy subjects or in different etiologies of FTR.

Our study patients did not undergo cardiac surgery and we could not therefore compare echocardiographic values vs anatomical/surgical measurements. The impact of TR on long-term mortality of patients has been well-known for many years [24]. In a large population of over 5000 patients from almost 20 years ago, patients with moderate or severe TR showed a higher mortality than patients with mild TR despite of pulmonary artery pressure and left ventricular ejection fraction [24]. These findings strongly suggest that increasing TR severity without surgical correction is associated with worse survival in the long term. In addition, failure of the annuloplasty procedure performed during a concomitant intervention result in later TR progression. Significant TR (3+ or 4+) was identified in 12% of patients (11/91) due to failure of TV repair or progression of untreated TR [25]. These findings support the clinical relevance of our study, in that part of surgical failures was due to suboptimal planning of annuloplasty based on 2D measurements under various filling conditions. However, it can be argued that size of the tricuspid ring is established directly in the operating room, though this occurs in para-physiological conditions, i.e., with a still and open heart with a totally empty right ventricle. Our study does not aim at replacing this direct measurement, which represents the cardiac surgeon’s final decision-making criterion for choosing the most appropriate ring, but simply aims at comparing 2D measurements with those obtained using the new 4D Auto TVQ tool. Our pivotal analysis highlighted the differences between the two methods in the RVD1, RVD2 and TV diameters measurements, in a sample where is showed a higher association of 4D measures and TR severity than 2D and TR severity.

## 5. Limitations

Many limitations to this retrospective study should be acknowledged. First, it was a single center, with a small number of patients. Multi-center studies on larger cohorts need to be recommended. Second, all echocardiographic datasets were acquired and measured by only one expert cardiologist and to achieve the interobserver variability cardiologists (fellows in echocardiography) with less experience were recruited. Third, our population was made by Caucasian volunteers only, which could limit the heterogeneity of our reference value. Fourth, our acquired TV measurements were mainly suitable to patients with high-quality echocardiographic images, which may restrict the generalizability of the results. However, currently there is no other commercially tool designed for TV quantification to compare our 3DE values. Moreover, we did not compare our measurements to CMR and CT results, but Muraru *et al*. [26] have tested the accuracy of their measurements obtained with 4D Auto-TVQ compared to cardiac CT but not to CMR imaging.

Further studies in this field with multi-center, larger cohorts of patients and control groups are warranted to validate these results.

## 6. Conclusions 

TA enlargement is the most important mechanism leading to FTR and the first therapeutic target for surgical and interventional repair procedures. The primary imaging modality to image the TV is 2DE, but its accuracy for quantitative analysis of TV apparatus is severely limited. The 4D Auto TVQ is a novel and modern tool for TV quantification that could be used as a complement to standard guideline-recommended 2DE. Specific patient subsets may benefit more from 4D Auto TVQ, including patients with functional TR in whom measurement of TA diameter and leaflet tethering can help identify the main mechanism of TR or patients with functional TR who are going to left-sided valve surgery and in whom the indication for TV repair is still unclear. So, a better knowledge of TV apparatus and FTR pathophysiology provides the basis for performing a personalized treatment approach, more accurate surgical planning, and effective surgical procedures.

## Availability of Data and Materials

Datasets used and/or analyzed for this study are available from the corresponding author upon appropriate request.

## References

[1] Fukuda S, Saracino G, Matsumura Y, Daimon M, Tran H, Greenberg NL (2006). Three-dimensional geometry of the tricuspid annulus in healthy subjects and in patients with functional tricuspid regurgitation: a real-time, 3-dimensional echocardiographic study. *Circulation*.

[2] Ton-Nu TT, Levine RA, Handschumacher MD, Dorer DJ, Yosefy C, Fan D (2006). Geometric determinants of functional tricuspid regurgitation: insights from 3-dimensional echocardiography. *Circulation*.

[3] Lancellotti P, Moura L, Pierard LA, Agricola E, Popescu BA, Tribouilloy C (2010). European Association of Echocardiography recommendations for the assessment of valvular regurgitation. Part 2: mitral and tricuspid regurgitation (native valve disease). *European Journal of Echocardiography: the Journal of the Working Group on Echocardiography of the European Society of Cardiology*.

[4] Vahanian A, Beyersdorf F, Praz F, Milojevic M, Baldus S, Bauersachs J (2022). 2021 ESC/EACTS Guidelines for the management of valvular heart disease. *European Heart Journal*.

[5] Rudski LG, Lai WW, Afilalo J, Hua L, Handschumacher MD, Chandrasekaran K (2010). Guidelines for the echocardiographic assessment of the right heart in adults: a report from the American Society of Echocardiography endorsed by the European Association of Echocardiography, a registered branch of the European Society of Cardiology, and the Canadian Society of Echocardiography. *Journal of the American Society of Echocardiography: Official Publication of the American Society of Echocardiography*.

[6] Lang RM, Badano LP, Mor-Avi V, Afilalo J, Armstrong A, Ernande L (2015). Recommendations for cardiac chamber quantification by echocardiography in adults: an update from the American Society of Echocardiography and the European Association of Cardiovascular Imaging. *Journal of the American Society of Echocardiography: Official Publication of the American Society of Echocardiography*.

[7] Writing Committee Members, Otto CM, Nishimura RA, Bonow RO, Carabello BA, Erwin JP (2021). 2020 ACC/AHA guideline for the management of patients with valvular heart disease: A report of the American College of Cardiology/American Heart Association Joint Committee on Clinical Practice Guidelines. *The Journal of Thoracic and Cardiovascular Surgery*.

[8] Addetia K, Muraru D, Veronesi F, Jenei C, Cavalli G, Besser SA (2019). 3-Dimensional Echocardiographic Analysis of the Tricuspid Annulus Provides New Insights Into Tricuspid Valve Geometry and Dynamics. *JACC. Cardiovascular Imaging*.

[9] Miglioranza MH, Mihăilă S, Muraru D, Cucchini U, Iliceto S, Badano LP (2015). Variability of Tricuspid Annulus Diameter Measurement in Healthy Volunteers. *JACC. Cardiovascular Imaging*.

[10] Knio ZO, Montealegre-Gallegos M, Yeh L, Chaudary B, Jeganathan J, Matyal R (2016). Tricuspid annulus: A spatial and temporal analysis. *Annals of Cardiac Anaesthesia*.

[11] Kjaergaard J, Petersen CL, Kjaer A, Schaadt BK, Oh JK, Hassager C (2006). Evaluation of right ventricular volume and function by 2D and 3D echocardiography compared to MRI. *European Journal of Echocardiography: the Journal of the Working Group on Echocardiography of the European Society of Cardiology*.

[12] Blusztein DI, Hahn RT (2023). New therapeutic approach for tricuspid regurgitation: Transcatheter tricuspid valve replacement or repair. *Frontiers in Cardiovascular Medicine*.

[13] Pizzino F, Trimarchi G, D’Agostino A, Bonanni M, Benedetti G, Paradossi U (2024). Impact of Leaflet-to-Annulus Index on Residual Regurgitation Following Transcatheter Edge-to-Edge Repair of the Tricuspid Valve. *Journal of Clinical Medicine*.

[14] Tanaka T, Sugiura A, Kavsur R, Vogelhuber J, Öztürk C, Becher MU (2022). Leaflet-to-annulus index and residual tricuspid regurgitation following tricuspid transcatheter edge-to-edge repair. *EuroIntervention: Journal of EuroPCR in Collaboration with the Working Group on Interventional Cardiology of the European Society of Cardiology*.

[15] Muraru D, Guta AC, Ochoa-Jimenez RC, Bartos D, Aruta P, Mihaila S (2020). Functional Regurgitation of Atrioventricular Valves and Atrial Fibrillation: An Elusive Pathophysiological Link Deserving Further Attention. *Journal of the American Society of Echocardiography: Official Publication of the American Society of Echocardiography*.

[16] Badano LP, Muraru D, Enriquez-Sarano M (2013). Assessment of functional tricuspid regurgitation. *European Heart Journal*.

[17] Muraru D, Hahn RT, Soliman OI, Faletra FF, Basso C, Badano LP (2019). 3-Dimensional Echocardiography in Imaging the Tricuspid Valve. *JACC. Cardiovascular Imaging*.

[18] Lancellotti P, Pibarot P, Chambers J, La Canna G, Pepi M, Dulgheru R (2022). Multi-modality imaging assessment of native valvular regurgitation: an EACVI and ESC council of valvular heart disease position paper. *European Heart Journal. Cardiovascular Imaging*.

[19] Min SY, Song JM, Kim JH, Jang MK, Kim YJ, Song H (2010). Geometric changes after tricuspid annuloplasty and predictors of residual tricuspid regurgitation: a real-time three-dimensional echocardiography study. *European Heart Journal*.

[20] Dreyfus GD, Raja SG, John Chan KM (2008). Tricuspid leaflet augmentation to address severe tethering in functional tricuspid regurgitation. *European Journal of Cardio-thoracic Surgery: Official Journal of the European Association for Cardio-thoracic Surgery*.

[21] Dreyfus GD, Martin RP, Chan KMJ, Dulguerov F, Alexandrescu C (2015). Functional tricuspid regurgitation: a need to revise our understanding. *Journal of the American College of Cardiology*.

[22] Dreyfus GD, Corbi PJ, Chan KMJ, Bahrami T (2005). Secondary tricuspid regurgitation or dilatation: which should be the criteria for surgical repair?. *The Annals of Thoracic Surgery*.

[23] Chikwe J, Itagaki S, Anyanwu A, Adams DH (2015). Impact of Concomitant Tricuspid Annuloplasty on Tricuspid Regurgitation, Right Ventricular Function, and Pulmonary Artery Hypertension After Repair of Mitral Valve Prolapse. *Journal of the American College of Cardiology*.

[24] Nath J, Foster E, Heidenreich PA (2004). Impact of tricuspid regurgitation on long-term survival. *Journal of the American College of Cardiology*.

[25] De Bonis M, Lapenna E, Sorrentino F, La Canna G, Grimaldi A, Maisano F (2008). Evolution of tricuspid regurgitation after mitral valve repair for functional mitral regurgitation in dilated cardiomyopathy. *European Journal of Cardio-thoracic Surgery: Official Journal of the European Association for Cardio-thoracic Surgery*.

[26] Muraru D, Gavazzoni M, Heilbron F, Mihalcea DJ, Guta AC, Radu N (2022). Reference ranges of tricuspid annulus geometry in healthy adults using a dedicated three-dimensional echocardiography software package. *Frontiers in Cardiovascular Medicine*.

